# The Chemokine Receptor CCR5, a Therapeutic Target for HIV/AIDS Antagonists, Is Critical for Recovery in a Mouse Model of Japanese Encephalitis

**DOI:** 10.1371/journal.pone.0044834

**Published:** 2012-09-21

**Authors:** Maximilian Larena, Matthias Regner, Mario Lobigs

**Affiliations:** 1 Department of Emerging Pathogens and Vaccines, John Curtin School of Medical Research, The Australian National University, Canberra, Australia; 2 Australian Infectious Diseases Research Centre, School of Chemistry and Molecular Biosciences, The University of Queensland, St Lucia, Australia; McMaster University, Canada

## Abstract

Japanese encephalitis is a severe central nervous system (CNS) inflammatory disease caused by the mosquito-borne flavivirus, Japanese encephalitis virus (JEV). In the current study we have investigated the immune responses against JEV in mice lacking expression of the chemokine receptor CCR5, which functions in activation and chemotaxis of leukocytes during infection. We show that CCR5 serves as a host antiviral factor against Japanese encephalitis, with CCR5 deficiency markedly increasing mortality, and viral burden in the CNS. Humoral immune responses, which are essential in recovery from JEV infection, were of similar magnitude in CCR5 sufficient and deficient mice. However, absence of CCR5 resulted in a multifaceted deficiency of cellular immune responses characterized by reduced natural killer and CD8^+^ T cell activity, low splenic cellularity, and impaired trafficking of leukocytes to the brain. Interestingly, adoptive transfer of immune spleen cells, depleted of B lymphocytes, increased resistance of CCR5-deficient recipient mice against JEV regardless of whether the cells were obtained from CCR5-deficient or wild-type donor mice, and only when transferred at one but not at three days post-challenge. This result is consistent with a mechanism by which CCR5 expression enhances lymphocyte activation and thereby promotes host survival in Japanese encephalitis.

## Introduction

The migration of leukocytes in lymphoid organs and to sites of inflammation is coordinated by an array of chemokines that bind to specific receptors on immune cells (reviewed in [1]). Of these, the chemokine receptor, CCR5, is expressed on natural killer (NK) cells, macrophages, and CD4^+^ and CD8^+^ T cells. In these cell types, it regulates chemotaxis and cell activation through interaction with the chemokine ligands CCL3, CCL4 and CCL5, which are up-regulated at the site of infection (reviewed in [2]). Understanding the role of CCR5 in the control of pathogen infections has important implications for human health beyond that of other chemokine ligand/receptor interactions, in view of the discovery that CCR5 is a major co-receptor for HIV-1 (reviewed in [3]). Therefore, the chemokine receptor is an important target for therapeutic intervention against HIV/AIDS, and recent clinical trials investigating the efficacy of CCR5 antagonists in patients with HIV/AIDS have provided promising results (reviewed in [4]). However, it has been argued that if CCR5 had a protective role against another group of pathogens, for instance in flaviviral encephalitis, it follows that a therapeutic treatment, which aims to block the receptor, could exacerbate the diseases caused by these pathogens [5], [6].

CCR5 was the first chemokine receptor recognized to play a critical role in recovery from flavivirus encephalitis in a study that showed that absence of CCR5 prevented efficient leukocyte trafficking to the brain and viral clearance in mice infected with West Nile virus (WNV) [7]. The important role of CCR5 in the human host response against West Nile encephalitis was supported by a retrospective cohort study involving persons homozygous for CCR5Δ32 [8], a loss-of-function mutation found in 1–2% of Caucasians [2]. Compared to individuals without the mutation, persons carrying a homozygous CCR5Δ32 allele had an increased risk for symptomatic WNV infection. This finding was corroborated with a large-scale database study, which associated homozygosity for CCR5Δ32 with an increased risk of early and late clinical manifestation following WNV infection [9]. CCR5Δ32 homozygosity has also been associated with severe tick-borne encephalitis symptoms [10] caused by infection with tick-borne encephalitis virus, and a severe case of yellow fever virus-associated viscerotropic disease [11]. Tick-borne encephalitis and yellow fever viruses are also members of the *Flavivirus* genus, raising the question of generality of CCR5 as an important host factor in recovery from flaviviral infections. Confirmation of a broader link between CCR5 deficiency and augmented incidence and severity of flaviviral disease would add to the concern of potential adverse outcomes associated with CCR5 antagonist use, in view of the large number of human infections inflicted by the different pathogenic members of the *Flavivirus* genus, and their widespread global distribution (reviewed in [12].

Here we have investigated the role and mechanism of CCR5 in recovery from infection in a mouse model of Japanese encephalitis. Japanese encephalitis virus (JEV) is closely related to WNV, and in terms of human disease incidence and severity the most important member of a serocomplex of mosquito-borne, encephalitic flaviviruses (reviewed in [13]). It is the leading cause of viral encephalitis in Asia, annually accounting for 30,000 to 50,000 cases and ∼10,000 deaths. Approximately 3 billion people in the Asia-Pacific region are at risk of infection with JEV. Host immune factors are thought to be the dominant determinants of disease outcome in Japanese encephalitis (reviewed in [14], [15]), with intact type I interferon and vigorous humoral immune responses essential for recovery, and CD8^+^ T cell immunity providing a subsidiary contribution to controlling JEV infection [16], [17]. With the use of CCR5-deficient mice we show in this study that the chemokine receptor is an additional important host factor involved in reducing disease severity with Japanese encephalitis.

## Materials and Methods

### Ethics statement

All animal experiments were approved by and conducted in accordance with the Australian National University (ANU) Animal Ethics Committee.

### Virus and cells

African green monkey kidney (Vero) cells were obtained from the American Type Culture Collection. Working stocks of JEV (strain Nakayama) were infected Vero cell culture supernatants (2×10^8^ PFU/ml) stored in single-use aliquots at −70°C. Vero cells and YAC-1 cells (Moloney leukemia virus-induced T cell lymphoma) [18] were grown at 37°C in Eagle's minimal essential medium plus nonessential amino acids (MEM) supplemented with 5% fetal bovine serum (FBS). Virus titration was by plaque assay on Vero cell monolayers, as described previously [19].

### Mice

Congenic CCR5^−/−^ mice (B6.129P2-Ccr5^tm1Kuz^/J) [20] and their wild-type controls were bred under specific-pathogen-free conditions and supplied by the Animal Breeding Facility at the John Curtin School of Medical Research, ANU, Canberra. Female mice were used in all experiments.

### Mouse inoculation and tissue collection

Mice were infected intravenously (i.v.) via the lateral tail vein with a single injection of 1×10^3^ PFU of JEV in 100 µl Hanks' balanced salt solution containing 20 mM HEPES (pH 8.0) and 0.2% bovine serum albumin (HBSS-BSA). Mice were monitored twice daily, and changes in 5 parameters including hair coat, posture, breathing pattern, activity, and movement were scored as normal (0), mild (1), moderate (2) or severe (3). Severely moribund mice were euthanized if the overall clinical score was >7, and/or when hindlimb paralysis was observed. For tissue processing, mice were euthanized at the time points indicated and a sterile midline vertical thoracoabdominal incision was made to expose the internal organs. After cardiac puncture for blood collection, animals were perfused with 10 ml sterile PBS. The brain and spinal cord were excised intact and collected for virus titration. For determination of virus titers, sample tissues were snap-frozen on dry ice. One-half of the brain samples were placed in cell culture medium and homogenized for lymphocyte isolation.

### Real-time RT-PCR

For determination of viral burden in mouse serum and spleen samples, total RNA in 50 µl splenic homogenates (10% [wt/vol]) and 50 µl serum was extracted using Trizol as described previously [21], and virion RNA content, expressed in genome equivalents, was determined by quantitative reverse transcription (RT)-PCR. For a genome copy standard, JEV RNA extracted from a Vero cell-grown virus stock and quantitated by spectrophotometry was used. RT was performed at 43°C for 90 min in a 10 µl mixture containing 2 µl sample RNA, Expand reverse transcriptase (Roche), RNase inhibitor (Invitrogen), 10 mM deoxynucleoside triphosphate, 10 pmol downstream primer (5′-TTGACCGTTGTTACTGCAAGGC-3′), 10 mM dithiothreitol, and the manufacturer's recommended buffer condition. Real-time PCR was performed using IQSybr qPCR mixture (Bio-Rad) and 0.2 nM downstream and upstream primers (5′-GCTGGATTCAACGAAAGCCACA-3′) under cycling conditions of 95°C for 3 min for 1 cycle and 95°C for 30 sec, 63°C for 30 sec and 72°C for 60 sec for 40 cycles. Each sample was tested in duplicate, and genome copy numbers were determined by extrapolation from a standard curve generated within each experiment. The detection limit of the assay was 4×10^3^ RNA copies/ml.

### Lymphocyte isolation from brain

Homogenized brain samples were digested with 2 mg/ml collagenase type I (Gibco-Life Technologies) in MEM plus 5% FBS for 45 min at 37°C with intermittent shaking and then centrifuged at 400× *g* for 10 min. Pellets were suspended in 2 ml 90% Percoll (GE Healthcare) in MEM plus 5% FBS and overlaid gently with 60%, 40% and 10% Percoll in MEM plus 5% FBS. The gradients were centrifuged at 800× *g* for 45 min at 25°C, and lymphocytes collected from the 40 to 60% interface were subsequently analyzed by flow-cytometry.

### Cell surface and intracellular cytokine staining

The surface marker staining employed in this study utilized the following reagents (all from Becton Dickinson except otherwise indicated): allophycocyanin (APC)-conjugated anti-CD8 antibody; phycoerythrin (PE)-conjugated anti-CD3, anti-NK1.1, anti-F4/80 (Caltag) and anti-CCR5 antibodies; fluorescein isothiocyanate (FITC)-conjugated anti-CD4, anti-NK1.1, anti-F4/80 (Serotec) and anti-CD19 antibodies; and peridinin-chlorophyll-protein complex (PerCP)-conjugated anti-CD3 antibody (BioLegend). 10^5^ events were acquired for each sample on a four-color FACSort flow-cytometer. Results were analyzed using Cell Quest Pro Software. For intracellular cytokine staining, 1×10^6^ splenocytes were suspended in 100 µl MEM with 5% FBS and stimulated for 16 h with 10^−4^ M H-2D^b^-binding 9-mer JEV NS4B protein-derived peptide, SAVWNSTTA [16], in the presence of 1 µl/ml brefeldin A (eBioscience). An ectromelia virus (ECTV) H-2K^b^-restricted peptide, TSYKFESV [22], was used at 10^−4^ M as a negative-control peptide. For stimulation of *ex vivo* splenocytes with JEV, splenocytes were infected at a multiplicity of infection (moi) of 1 for 1 h at 37°C and washed twice with MEM containing 5% FBS before incubation for 16 h in MEM plus 5% FBS in the presence of brefeldin A. Enumeration of activated NK cells by intracellular staining for IFN-γ expression was performed directly on ex vivo splenocytes. Cells were surface stained with anti-CD8–APC or anti-NK1.1-PE antibodies before paraformaldehyde fixation and permeabilization with saponin (Biosource) according to the supplier's instruction. Cells were then stained with anti-IFN-γ–FITC (BioLegend) and/or anti-tumor necrosis factor alpha (TNF-α)–PE (Invitrogen), and washed twice with fluorescence-activated cell sorter (FACS) washing buffer (2% FBS in PBS) before assessment by FACS analysis.

### NK cell cytotoxic assay

Mice were infected with 10^3^ PFU of JEV, i.v., and sacrificed at days 4 and 7 post-infection (pi). Spleens were collected and tested for NK cell cytotoxicity in a standard ^51^Cr-release assay [23], using uninfected YAC-1 cells as targets. Splenocytes from uninfected mice were used as controls. P values were calculated from a four-point logarithmic regression curve, interpolated at an effector-to-target (e/t) cell ratio of 30.

### Transfer experiments

Eight-week-old CCR5^−/−^ or CCR5^+/+^ mice were infected with 1×10^3^ PFU of JEV, i.v., and sacrificed a week later for aseptic removal of spleens. Single-cell splenocyte suspensions were prepared by pressing the spleen tissue gently through a fine metal mesh tissue sieve. Erythrocyte lysis was by suspension of the splenocyte pellet in 4.5 ml distilled water, followed immediately by the addition of 0.5 ml of 10× PBS. Lysed cells were discarded after centrifugation at 400×*g* for 5 min. Splenocytes were resuspended in 100 µl PBS and injected through the lateral tail vein into 8-week-old CCR5^−/−^ recipient mice. Recipient mice were challenged one or three days later with 1×10^3^ PFU JEV via footpad injection. For B cell depletion, splenocytes were incubated with anti-CD19 magnetic beads (Miltenyi Biotec) and loaded onto magnetic columns according to the supplier's instructions. Effluent from the columns was collected, and cells were pelleted by centrifugation at 400×*g* for 5 min. The efficiency of B cell depletion was 99% as assessed by FACS analysis.

### Serological tests

For titration of JEV-specific antibody isotypes in mouse serum, ELISAs were performed with HRP-conjugated rabbit anti-mouse IgM, IgG1 and IgG2b (Serotec), and the peroxidase substrate 2,2′-azino-di(3-ethyl-benzthiazoline sulfonate). The JEV Nakayama strain was used for ELISA antigen production as described previously for Murray Valley encephalitis virus [24]. For determination of ELISA endpoint titers, absorbance cut-off values were established as the mean absorbance of eight negative-control wells containing sera of naive mice plus 3 standard deviations (SD). Absorbance values of test sera were considered positive if they were equal to or greater than the absorbance cut-off, and endpoint titers were calculated as the reciprocal of the last dilution giving a positive absorbance value. Neutralization titers, measured in a 50% plaque reduction neutralization test (PRNT_50_), were determined as previously described [25].

## Results

### CCR5 is required for protection from Japanese encephalitis

**Figure 1:**
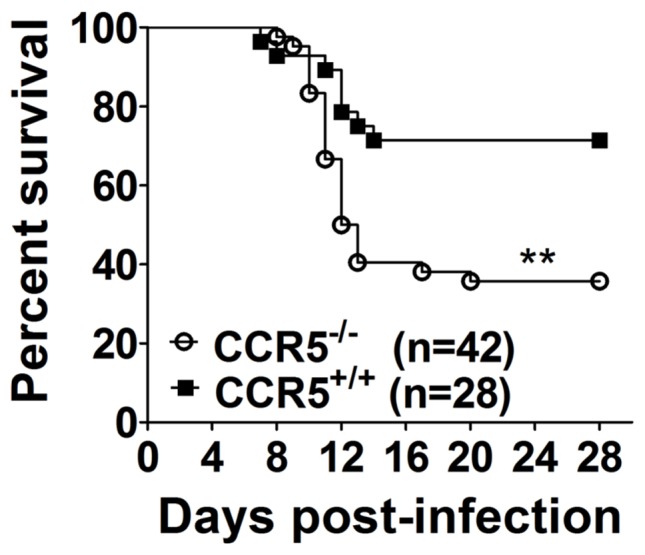
Susceptibility of CCR5^+/+^ and CCR5^−/−^ mice to infection with JEV. Groups of 8-week-old mice were infected i.v. with 10^3^ PFU of JEV. Morbidity and mortality were recorded daily, and surviving mice were monitored for 28 days. The data shown were constructed from two independent experiments. The significance of differences in mortality between wild-type (n = 28) and knockout mice (n = 42) was determined by using the log-rank test (**, *P*<0.01).

To assess the impact of CCR5 on the pathogenesis of Japanese encephalitis, mortality was recorded in 8-week-old congenic CCR5^−/−^ and CCR5^+/+^ wild-type mice after i.v. challenge with 10^3^ PFU of JEV. Moribund mice in both groups presented with similar clinical signs starting with generalized piloerection, paresis and rigidity, invariably progressing to severe neurological signs demonstrated by postural imbalance, ataxia and generalized tonic-clonic seizures. Median survival time amongst mice that succumbed to infection did not differ between the two groups (11.7±2.3 days for CCR5^−/−^ and 11.1±2.1 days for CCR5^+/+^ mice). Nevertheless, absence of CCR5 resulted in a significant increase in mortality: 64% in CCR5^−/−^ versus 28% in CCR5^+/+^ mice (**Figure 1**).

**Figure 2:**
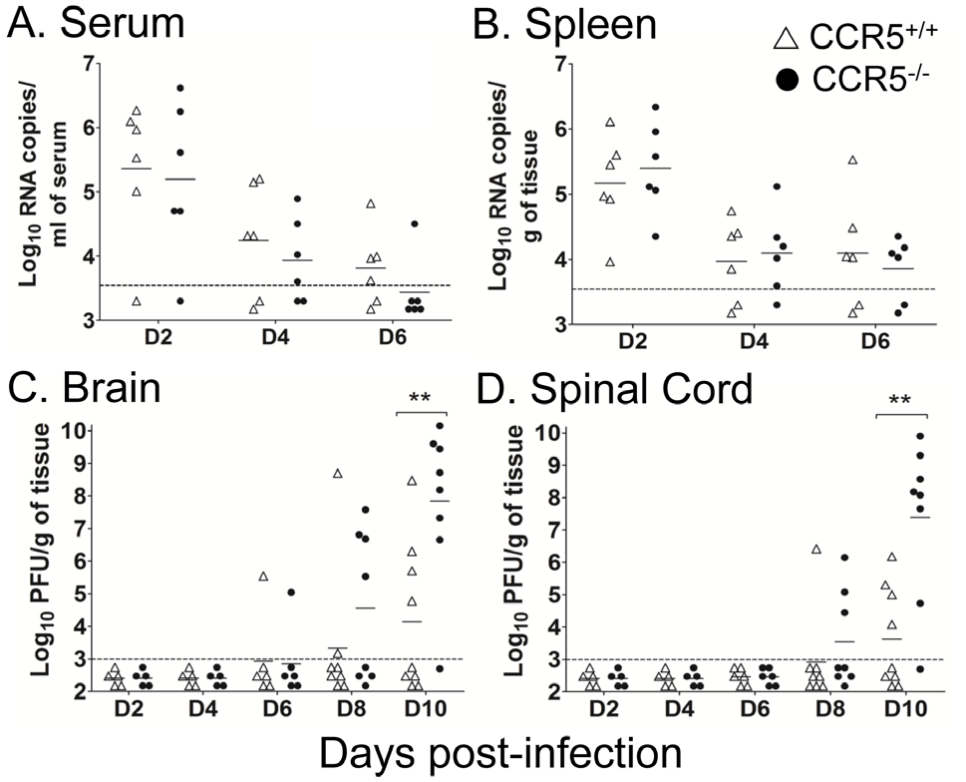
JEV burden in serum and tissue samples. JEV burden in (**A**) serum, (**B**) spleen, (**C**) brain and (**D**) spinal cord of CCR5^+/+^ and CCR5^−/−^ mice after i.v. infection with 10^3^ PFU of JEV. At the indicated time points, animals were sacrificed and the viral RNA content of serum and spleen samples were measured by real-time RT-PCR, while viral content in brain and spinal cord samples were measured by plaque titration. Data shown were constructed from 2 independent experiments. Each symbol represents an individual mouse, and geometric mean titers are indicated by horizontal lines. The lower limit of virus detection is indicated by the horizontal dotted line. Asterisks denote significant differences (**, *P*<0.01).

To address the mechanism for increased susceptibility of mice to JEV infection in the absence of CCR5, viral titers in CCR5^−/−^ and control mice infected with 10^3^ PFU, i.v., were determined by real-time RT-PCR in serum and spleen, and by plaque assay in brain and spinal cord. Both groups displayed similar viral burden in serum from day 2 to day 6 pi, with peak viremia occurring on day 2 pi (**Figure 2A**). Similarly, viral load in spleen did not differ significantly between the two groups (**Figure 2B**). On the other hand, viral spread into and/or clearance from the CNS was significantly affected by the absence of CCR5: compared to control mice, viral load in brains of CCR5^−/−^ mice was between 10- and 10,000-fold higher on days 8 and 10 pi, respectively, with the proportion of mice showing detectable virus titers markedly greater in CCR5^−/−^ mice than that in the control group (**Figure 2C**). In addition, viral spread in the CNS was more pronounced in CCR5^−/−^ mice, with viral titers in spinal cord exceeding those in CCR5^+/+^ mice by four log on day 10 pi (**Figure 2D**).

Together, these data suggest that CCR5 is critically required for recovery from JEV infection.

### Humoral immunity in the absence of CCR5

**Figure 3:**
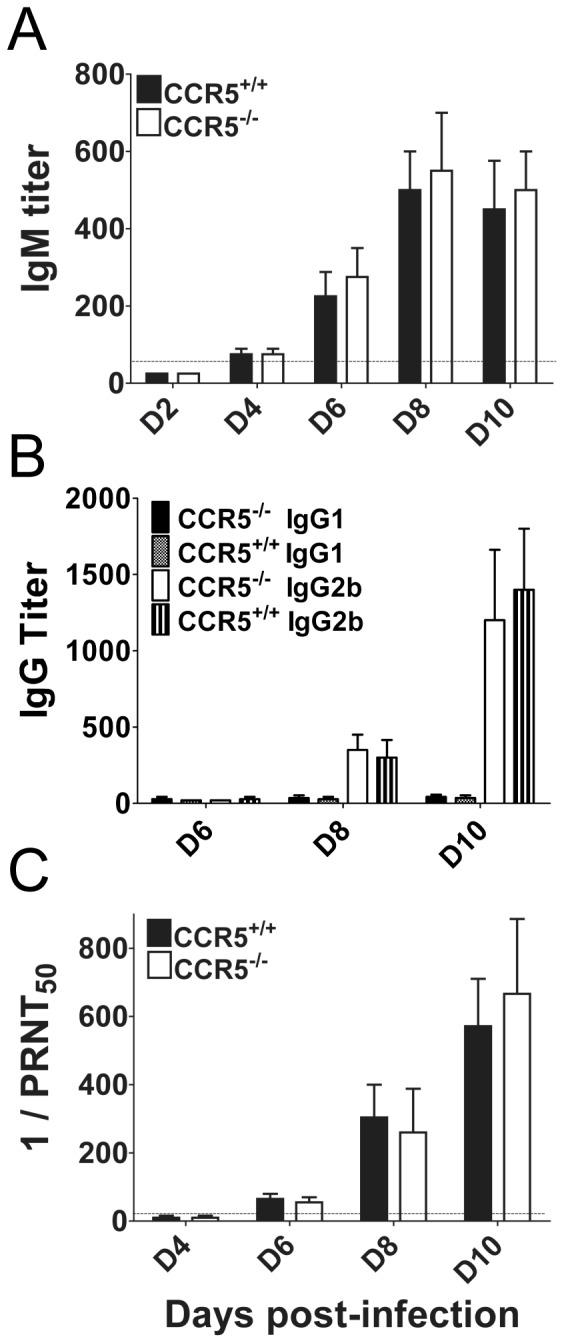
Antibody responses in CCR5^+/+^ and CCR5^−/−^ mice. Eight-week-old CCR5^+/+^ and CCR5^−/−^ mice were infected i.v. with 10^3^ PFU of JEV, and serum samples were collected at the indicated time points. Anti-JEV IgM (**A**) and IgG (**B**) isotype antibody titers were determined by ELISA. The data presented are reciprocal mean endpoint titers representative of 4 mice per time point with the SEM indicated by error bars. (**C**) Neutralizing antibody titers determined by plaque reduction neutralization assay. The data presented are mean PRNT_50_ titers representative of 5 mice per time point, and error bars indicate the SEM.

We have previously established a pivotal role of antibody in recovery from JEV infection [16]. Given that CCR5 is expressed on CD4^+^ T cells, which are required for efficient generation and maintenance of humoral immunity against JEV, we investigated whether a deficiency in CCR5 would result in poorer JEV-specific antibody responses. However, the kinetics and magnitude of the IgM response was similar in CCR5^−/−^ and control mice, appearing first on day 4 pi, and peaking on day 8 pi (**Figure 3A**). Class switching to IgG production was also comparable between both groups, initially detected on day 8 pi, and increasing on day 10 pi (**Figure 3B**). Assessment of functional activity of the antibody responses by neutralization assay revealed no significant difference between the two groups, reaching mean PRNT_50_ titers of 660 (range, 80 to 1280) in CCR5^−/−^ mice and 570 (range, 160 to 1280) in CCR5^+/+^ mice on day 10 pi (**Figure 3C**).

These data indicate that a deficiency in CCR5 does not compromise the ability of mice to prime JEV-specific B cell immune responses.

### Blunted NK and CD8^+^ T cell responses in CCR5^−/−^ mice

**Figure 4:**
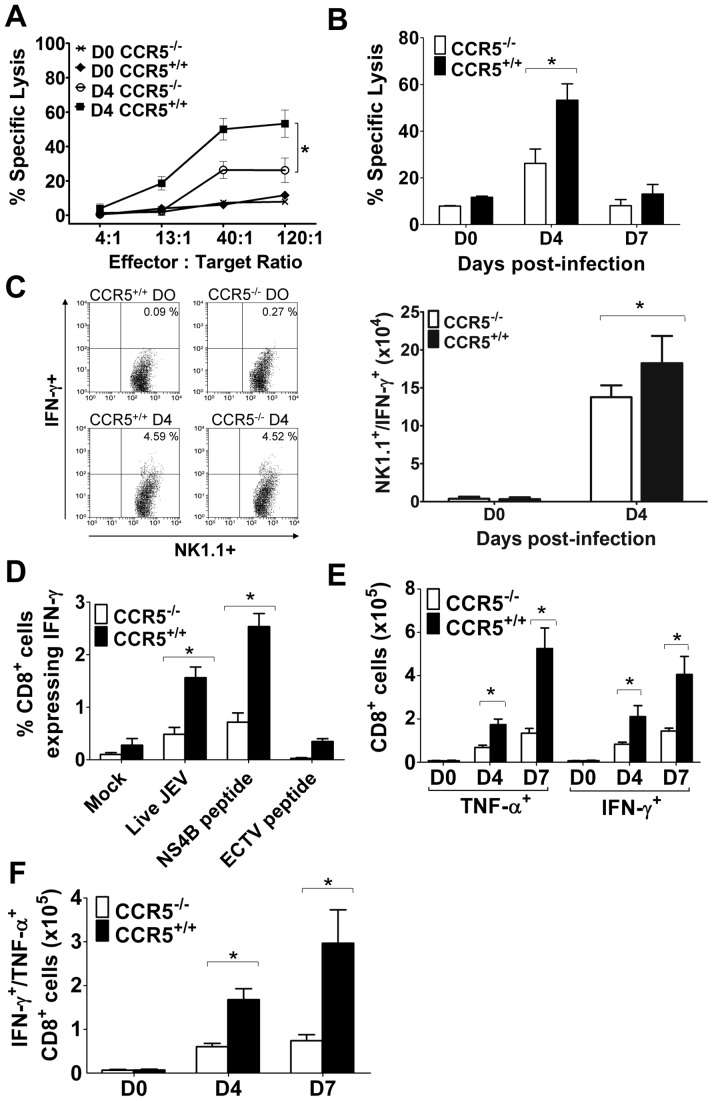
NK and CD8^+^ T cell responses in CCR5^+/+^ and CCR5^−/−^ mice infected with JEV. Eight-week-old CCR5^+/+^ and CCR5^−/−^ mice were infected i.v. with 10^3^ PFU of JEV or left uninfected. (**A**) Spleens were collected at day 4 pi and tested for NK cell cytotoxicity in a standard ^51^Cr release assay using uninfected YAC-1 cells as targets. The percentage of NK cell lysis is plotted against an increasing e/t cell ratio. Means for 5 samples ± SEM are presented, and data are representative of 2 independent experiments. (**B**) NK cell cytotoxicity at indicated time points with an e/t cell ratio of 120∶1. Means for 5 samples ± the SEM are presented, and data are representative of 2 independent experiments. (**C**) Numbers of NK1.1^+^ stained splenocytes expressing IFN-γ were identified by flow cytometry at day 0 and 4 p.i. Means for 5 samples ± the SEM are presented, and data are representative of 2 independent experiments. (**D**) Splenocytes harvested at day 7 p.i. and stimulated *ex vivo* with live JEV, H-2D^b^-restricted JEV NS4B protein-derived peptides, H-2D^b^-restricted ECTV negative-control peptide or mock treated, and IFN-γ production in CD8^+^ T cells was measured by flow cytometry. Means for 3 samples ± the SEM are presented, and data are representative of 2 independent experiments. (**E**) Kinetics of JEV immune CD8^+^ T cell activation measured after *ex vivo* stimulation with D^b^-restricted JEV NS4B peptide, showing percentage of CD8^+^ T cells per spleen that express IFN-γ, TNF-α, or (**F**) both cytokines following stimulation. Asterisks denote significant differences (*, *P*<0.05).

NK cells form an important part of the cellular arm of the host's innate immunity, and function by killing virally infected cells and by release of the cytokines, IFN-γ and TNF-α. NK cell activity in JEV infected CCR5^−/−^ and control mice were determined by measuring cytolytic activity against YAC-1 cells and by intracellular staining of IFN-γ. At the peak of NK cell activity (day 4 pi), lysis of YAC-1 cells was significantly reduced in CCR5^−/−^ mice compared to CCR5^+/+^ mice (**Figure 4A and 4B**). This was substantiated by a significantly reduced number of activated, IFN-γ-expressing, NK cells in spleen of CCR5^−/−^ relative to control mice at day 4 pi (**Figure 4C**).

CD8^+^ T cells form the dominant cytotoxic arm of the adaptive immune system. We previously reported a subsidiary role of CD8^+^ T cells in recovery from JEV infection [16], and hypothesized that a defective CD8^+^ T cell response could, at least in part, account for the increase in susceptibility of CCR5^−/−^ mice to JEV infection. The primary murine CD8^+^ T cell responses against JEV and other flaviviruses peak at 7 days pi, and thereafter markedly decline [16], [26], [27]. **Figure 4D** demonstrates a significantly blunted JEV-immune CD8^+^ T cell response in CCR5^−/−^ relative to control mice, determined by intracellular cytokine staining following ex vivo stimulation of CD8^+^ lymphocytes with a JEV NS4B protein-derived H-2D^b^-binding peptide. Relative to CCR5^+/+^ mice, CCR5^−/−^ mice showed a 61% and 74% reduction in the number of IFN-γ-secreting CD8^+^ T cells, and a 60% and 64% reduction in the number of TNF-α-secreting CD8^+^ T cells in spleen on days 4 and 7 pi with JEV, respectively (**Figure 4E**). This was also reflected in a significantly smaller number of dual-functional JEV-immune CD8^+^ T cells (defined as the number of CD8^+^ T cells expressing both IFN-γ and TNF-α following stimulation with cognate peptide), which was reduced by 3-fold and 4-fold on days 4 and 7 pi, respectively, in the absence of CCR5 expression (**Figure 4F**).

Collectively, these data indicate that a deficiency in CCR5 results in blunted NK and CD8^+^ T cell responses after JEV infection.

### Impaired leukocyte proliferation and trafficking in CCR5^−/−^ mice

**Figure 5:**
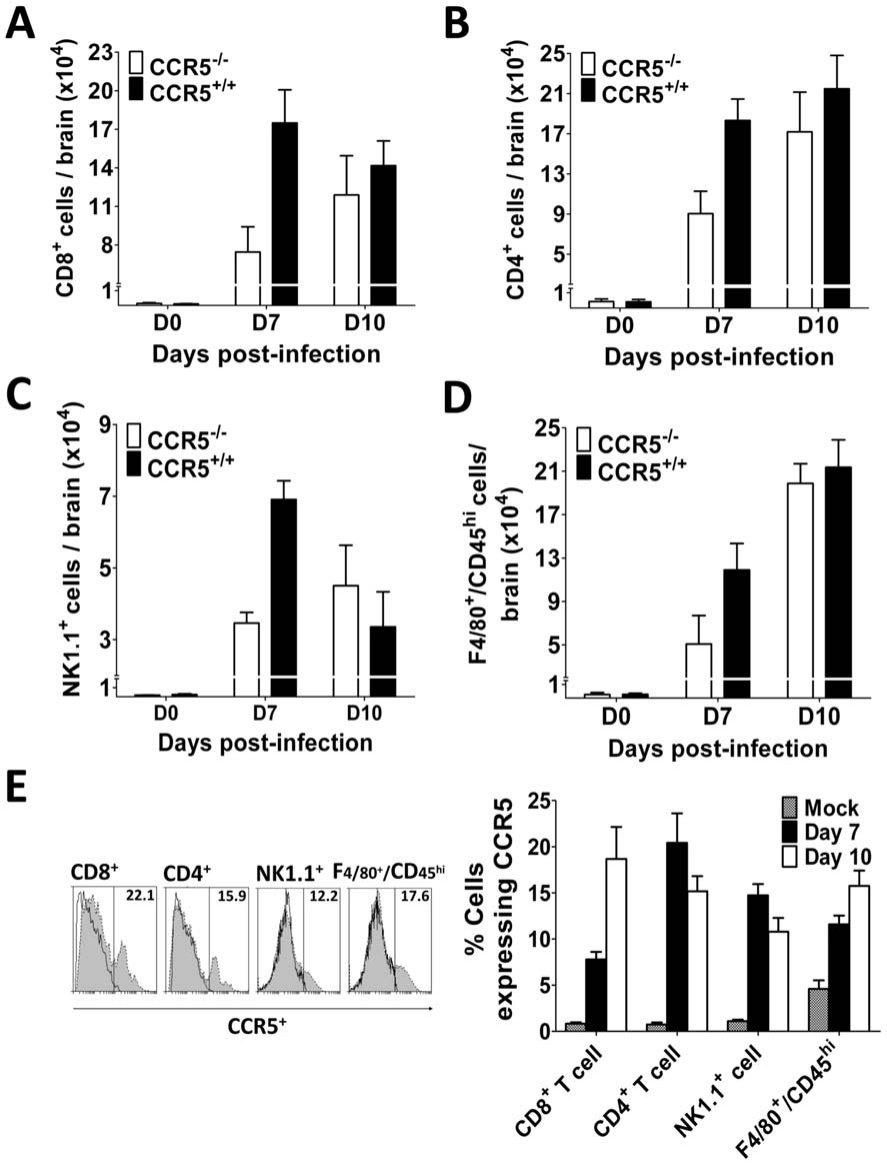
Leukocyte trafficking into the CNS. Kinetics of cell infiltration into the brain of 8-week-old CCR5^+/+^ and CCR5^−/−^ mice infected i.v. with 10^3^ PFU of JEV. Leukocytes were isolated at indicated time points from the brains of infected mice and stained for identification as (**A**) CD8^+^ T cell, (**B**) CD4^+^ T cell, (**C**) NK1.1^+^ cell and (**D**) F4/80^+^/CD45^Hi^ infiltrating macrophages. (**E**) Leukocyte subpopulations were gated for CCR5 expression; histograms show CCR5 expression on brain-derived leukocyte subpopulations on day 10 pi (shaded histograms). Means are derived from 4–9 samples per time point, error bars denote the SEM, and data are representative of 2 independent experiments. Asterisks denote significant differences (*, *P*<0.05; **, *P*<0.01).

CCR5 expression drives the migration of leukocytes into the CNS after WNV infection, and thereby contributes to viral clearance and recovery from infection [7]. To investigate whether this is also evident in our mouse model of Japanese encephalitis, we isolated leukocytes from brains of JEV infected CCR5^+/+^ and CCR5^−/−^ mice by density gradient centrifugation and quantified leukocyte subpopulations by flow-cytometry. On day 7 pi, CCR5^−/−^ mice showed a significant (∼50%) reduction in infiltration of leukocytes into the brain relative to control mice, and this effect was found for all CCR5-expressing subpopulations, viz. NK cells, F480^+^/CD45^hi^ macrophages, CD8^+^ T cells and CD4^+^ T cells (**Figure 5A–D**). This difference ceased to be significant by day 10 pi. These data show that in the case of Japanese encephalitis, the absence of CCR5 resulted in delayed trafficking of leukocytes into the brain, despite the much higher virus titers in the CNS of infected CCR5^−/−^ than CCR5^+/+^ mice.

Given the role of CCR5 expression in augmented leukocyte trafficking into the brain, we next assessed the proportion of cells of the different leukocyte subpopulations that express the chemokine receptor using CCR5^+/+^ mice that were infected with JEV. On day 7 pi, 15% of NK cells, 12% of F4/80^+^/CD45^hi^ infiltrating macrophages, 8% of CD8^+^ T cells and 20% of CD4^+^ T cells were CCR5-positive (**Figure 5E**). This proportion increased on day 10 pi in the case of CD8^+^ T cells (19%) and F4/80^+^/CD45^hi^ infiltrating macrophages (16%), but decreased for NK and CD4^+^ T cells. Similar numbers of infiltrating CCR5-positive cells as a proportion of the different leukocyte subpopulations in the brain have been observed in mice with West Nile encephalitis [7].

**Figure 6:**
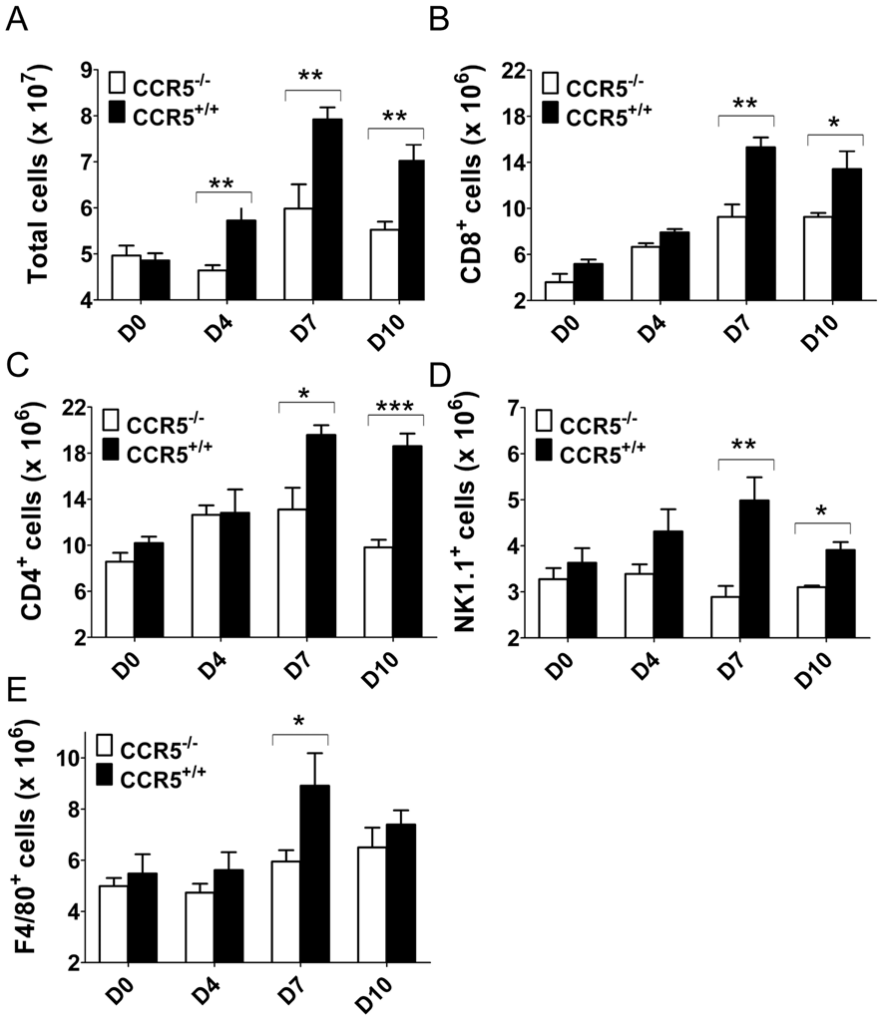
Leukocyte numbers in spleen after JEV infection. Kinetics of cell expansion in spleens of 8-week-old CCR5^+/+^ and CCR5^−/−^ mice infected i.v. with 10^3^ PFU of JEV. (**A**) Spleens were collected at indicated time points from infected mice and total splenocyte numbers were determined. Cells were then stained and identified as CD8^+^ T cell (**B**), CD4^+^ T cell (**C**), NK1.1^+^ cell (**D**) and F4/80^+^ macrophages (**E**). Means are derived from 4–8 samples per time point, error bars denote the SEM, and data are representative of 2 independent experiments. Asterisks denote significant differences (*, *P*<0.05; **, *P*<0.01; ***, *P*<0.001).

To assess whether impaired trafficking of leukocytes, *per se*, or a failure of leukocyte expansion in the periphery accounted for the reduced numbers of immune cells in the brain of infected CCR5^−/−^ relative to control mice, we quantified splenic cellularity in CCR5^−/−^ and CCR5^+/+^ mice at various time points after JEV infection. As expected, given that spleen serves as the main immune-responsive lymphoid organ after an i.v. challenge, total splenocyte numbers in CCR5^+/+^ mice increased following infection with JEV, with the peak expansion on day 7 pi (8×10^7^ cells/spleen; **Figure 6A**). This contrasted with a significantly lesser number of splenocytes in JEV infected mice lacking CCR5 expression, although the kinetics of the response was similar between the two groups of mice (**Figure 6A**). The deficit in cell numbers was found across the different CCR5-encoding splenocyte subpopulations, showing a reduction of 43% for NK cells, 33% for F4/80^+^ macrophages, 34% for CD4^+^ T cells, and 39% for CD8^+^ T cells in CCR5^−/−^ mice relative to control animals on day 7 pi (**Figure 6B–E**). The percentage reduction of T cell numbers and activation phenotype (**Figure 4**) in spleen on day 7 pi correlated closely with the differential leukocyte migration into the brains of CCR5^−/−^ and wt mice (**Figure 5**).

Together these data imply that the impaired trafficking of leukocytes into the CNS after JEV infection in CCR5^−/−^ mice may, at least in part, be a direct result of a deficit in leukocyte numbers in the periphery.

### Early transfer of CCR5-deficient splenocytes increases survival of recipient CCR5^−/−^ mice challenged with JEV

Our mechanistic studies on the role of CCR5 in recovery from JEV infection showed deficiencies in leukocyte trafficking to the CNS, blunted NK and CD8^+^ T cell responses, and low splenic cellularity, while a previous investigation on WNV concluded that CCR5 increased host survival predominantly by promoting leukocyte migration to the infected brain [7]. To test whether defective immune cell priming and expansion significantly contributed to the increased susceptibility of CCR5^−/−^ mice to JEV infection, we adoptively transferred splenocytes from JEV infected CCR5^−/−^ and CCR5^+/+^ mice to CCR5^−/−^ recipients lethally challenged with the virus. Complete protection was achieved when total immune splenocytes from either donor strain were transferred at day 1 pi with JEV (**Table 1**). In contrast, no increase in survival was observed when the transfers were performed at 3 days pi, independent of whether the donor mice were CCR5 sufficient or deficient. This suggested that following the establishment of a CNS infection, viral clearance and protection mediated by the adaptive immune responses is ineffective in the case of Japanese encephalitis. This conclusion is not limited to the CCR5-deficient recipient mice, since a similar lack of protective value against JEV of late transfer (day 3 post-challenge) of immune spleen cells was also found in wild-type B6 recipients (unpublished data).

**Table 1:** Adoptive transfer of immune splenocytes from CCR5^+/+^ or CCR5^−/−^ donor mice protect CCR5-deficient recipient mice against Japanese encephalitis.

Treatment^a^	Mortality (No. of deaths/total)^b^	Mean survival time (days) ± SD^c^
PBS control	71% (15/21)	11.4±1.5
Transfer at one day post-challenge		
Naïve CCR5^+/+^ splenocytes (1×10^7^ cells)	67% (8/12; *P* = 0.92)	11.3±2.3
Naïve CCR5^−/−^ splenocytes (1×10^7^ cells)	78% (10/14; *P* = 0.93)	11.6±2.2
Immune CCR5^+/+^ splenocytes (1×10^7^ cells)	0% (0/10; *P* = 0.0006)	-
Immune CCR5^−/−^ splenocytes (1×10^7^ cells)	0% (0/10; *P* = 0.0006)	-
Immune CCR5^+/+^ splenocytes, B cell-depleted (5×10^6^ cells)	33% (2/9; *P* = 0.019)	12.0±2.8
Immune CCR5^−/−^ splenocytes, B cell-depleted (5×10^6^ cells)	20% (2/10; *P* = 0.012)	11.5±0.7
Transfer at three days post-challenge		
Immune CCR5^+/+^ splenocytes (2×10^7^ cells)	67% (6/9; *P* = 0.57)	12.2±1.9
Immune CCR5^−/−^ splenocytes (2×10^7^ cells)	80% (8/10; *P* = 0.65)	11.8±2.8

a Eight-week-old donor CCR5^+/+^ or CCR5^−/−^ mice were infected with 10^3^ PFU of JEV, i.v., or left uninfected and sacrificed 7 days later for splenocyte collection with or without depletion of B cells. Cells were transferred to 8-week-old CCR5^−/−^ recipient mice infected 3 days or 1 day earlier with 10^3^ PFU of JEV i.v. Surviving mice were monitored for 28 days.

b Data are representative of 2 independent experiments. Immune splenocyte treatment groups were compared to the naive splenocyte control group to test for statistical significance.

c No significant difference was noted between immune splenocyte treatment and control groups.

Given the dominant role of B cells (which do not express CCR5) in recovery from Japanese encephalitis [16], we next performed adoptive transfer experiments of B cell-depleted splenocytes from CCR5^−/−^ and CCR5^+/+^ donor mice to CCR5^−/−^ recipients at one day after JEV infection. Interestingly, even B cell-depleted splenocytes from both donor mice provided significant, albeit not complete, protection, increasing the survival rate of recipient mice to that of untreated CCR5^+/+^ mice infected with JEV (**Table 1**).

Together, these data confirm the important role of B cells in protection against Japanese encephalitis, and indicate that CCR5 expression was not required for the disease-ameliorating effect of B cell-depleted lymphocytes primed against JEV. The data also suggest that in adoptive transfer of immune T cells control of JEV infection likely occurred extraneurally, since protection was seen only when the cells were provided early pi, and that accordingly migration of the lymphocytes to peripheral sites of infection did not require expression of CCR5.

## Discussion

The role of the chemokine receptor CCR5 in infection is variable in terms of its impact on pathogenesis and disease outcome. An important disease-ameliorating contribution of CCR5 has been documented in settings of trypanosomiasis [28], toxoplasmosis [29], influenza [30], [31], parainfluenza [32], West Nile encephalitis [7], tick-borne encephalitis [10], genital herpes [33], and chlamydia infection [34], while no significant effect of CCR5 deficiency on the outcome of other microbial infections could be found [35]–[39]. Mechanistically, the protective value of CCR5 has been largely attributed to its regulatory effect on leukocyte trafficking to the site of infection, although additional functions of CCR5 in the immune response involving antigen recognition [36], [40], [41], priming and proliferation of lymphocytes [33], [36], [37], [40]–[44], and T cell memory formation [32], [45] have also been recognized. In contrast, a disease-potentiating effect of CCR5 has been found in experimental cerebral malaria [46], schistosomiasis [47], leishmaniasis [48], cryptococcosis [49], herpes keratitis [50], mouse hepatitis virus-induced multiple sclerosis [51], and HIV/AIDS [52], mostly as a consequence of increased immunopathology, and in the case of HIV/AIDS due to usage of CCR5 as an alternative receptor for virus infection (reviewed in [3]). This dichotomy in the role of CCR5 in the immune response to infection with different pathogens prevents generalization of the impact of the chemokine receptor in disease prognosis.

In the present study we establish a critical role of CCR5 in recovery from Japanese encephalitis, which was reflected in increased viral burden in the CNS, but not extraneural tissues, and increased mortality in the absence of CCR5 expression. It was previously shown that type I interferon and antibody, but not T cells, are key to control of virus replication in peripheral tissues in the mouse model of Japanese encephalitis, while T cells play a role in reducing viral burden and dissemination in the CNS [16]. CCR5-deficient mice displayed a wide-ranging debilitation of JEV-specific immune responses; these included poor NK cell activity, a suboptimal JEV-immune CD8^+^ T cell response, low splenic cellularity, and impaired trafficking of leukocytes (NK cells, macrophages, and CD8^+^ and CD4^+^ T cells) to the brain. These factors most likely acted in concert to create a lethal combination of defective viral clearance from peripheral tissues and the CNS, thereby increasing the susceptibility of the host to JEV infection. In addition, recent findings suggest that CCR5, which is expressed in neurons and is up-regulated in the brain in pathological conditions, could have a direct neuro-protective function by increasing neuronal survival (reviewed in [2]).

Our results are consistent with the previous finding of increased susceptibility of CCR5-deficient mice in a model of West Nile encephalitis [7], although mechanistic differences in the role of CCR5 in recovery from WNV and JEV infection are apparent. While a significant (≥50%) overall reduction in migration of NK cells, CD4^+^ and CD8^+^ T cells, and activated infiltrating macrophages to the brain of JEV and WNV infected CCR5^−/−^ mice was seen in both investigations, this difference relative to CCR5^+/+^ control mice narrowed between days 7 and 10 pi in the case of JEV infection, but became more pronounced for WNV. More importantly, the WNV study found similar splenic immune responses in CCR5 sufficient and deficient mice, whereas we observed markedly reduced numbers of lymphocytes in spleens of mice lacking CCR5 expression. It is likely that the latter was a major factor contributing to the severe phenotype in JEV infected CCR5-deficient mice, since adoptive transfer of B cell-depleted immune splenocytes corrected the phenotype, independent of whether the cells were derived from CCR5^−/−^ or CCR5^+/+^ donor mice. Low splenocyte numbers in the absence of CCR5 may reflect restricted leukocyte proliferation secondary to poor early priming, increased cell turnover to other lymphoid organs, and/or increased T cell death. However, since the spleen is the main responsive peripheral lymphoid organ following an i.v. challenge, defective proliferation most likely accounts for the diminished splenocyte numbers in CCR5−/− mice. Moreover, other studies have also observed restricted proliferation of splenocytes, or specific subsets thereof, in the absence of CCR5, both in physiologic [53] and pathologic conditions [33], [36]. The latter studies deal with virus challenge models with a resultant impairment in control of infection.

Concomitant with the limited number of leukocytes in spleens of CCR5-deficient mice infected with JEV, the functional activities of NK and CD8^+^ T cell responses were blunted, while the antibody response against JEV did not significantly differ from that elicited in infected wild-type mice. This finding was consistent with several other studies that demonstrated a critical role of CCR5 expression on naïve T cells in activation of CD8^+^ T cell responses by guiding the cells to and enhancing interaction with antigen-presenting dendritic cells in immunogen-draining lymph nodes [40]–[44]. We have previously found that co-transfer of immune CD4^+^ and CD8^+^ T cells, but not individual transfer of either lymphocyte subpopulations, was protective in lethal infection with JEV [16]. Thus, the suboptimal stimulation of virus-specific T cell responses together with their reduced migration into the CNS most likely accounted for the increased susceptibility of CCR5-deficient mice to Japanese encephalitis.

It is unclear whether the reduced NK cell activity in CCR5-deficient relative to wild-type mice contributed to the increased disease severity with Japanese encephalitis, as has been proposed in studies on herpes simplex virus in CCR5^−/−^ mice [33], [36]. Flaviviruses are in general poor inducers of NK cells, and flavivirus infection reduces the susceptibility of target cells to NK cell lysis [23], [54]. The latter is thought to be a consequence of virus-induced up-regulation of MHC-I on the surface of flavivirus-infected cells [55]–[58]. Therefore, it appears that NK cell responses do not markedly enhance recovery from flavivirus infection. Consistent with this interpretation, in vivo depletion of NK cells using monoclonal antibody anti-NK1.1 did not result in increased mortality or increased viral burden in the CNS in adult mice infected with JEV (our unpublished result) or WNV [59].

In conclusion, our study established CCR5 as a host factor against severe disease in a mouse model of Japanese encephalitis. Control and elimination of viral spread is largely dependent on rapid activation of antiviral leukocytes, and their recruitment to the sites of peripheral infection and/or into the CNS. In the absence of CCR5, an extensive immune dysfunction ensued, encompassing probable involvement of defective early priming and subsequent aberrant leukocyte activation, restricted splenocyte expansion, and limited trafficking. This finding raises possible safety concerns regarding the use of CCR5 antagonists in HIV individuals, particularly in regions where both HIV and JEV are endemic [60], [61]. While the benefits of taking the drug most likely far outweigh the potential risk of enhanced susceptibility to Japanese encephalitis, mitigating the latter risk factor by instituting precautionary measures including patient education, limiting mosquito exposure, and/or vaccination may be warranted.

## References

[1] Allen SJ, Crown SE, Handel TM (2007) Chemokine: receptor structure, interactions, and antagonism. Annu Rev Immunol 25: 787–820. 17291188 10.1146/annurev.immunol.24.021605.090529

[2] Sorce S, Myburgh R, Krause KH (2011) The chemokine receptor CCR5 in the central nervous system. Prog Neurobiol 93: 297–311. 21163326 10.1016/j.pneurobio.2010.12.003

[3] Alkhatib G (2009) The biology of CCR5 and CXCR4. Curr Opin HIV AIDS 4: 96–103. 19339947 10.1097/COH.0b013e328324bbec PMC2718543

[4] Wilkin TJ, Gulick RM (2012) CCR5 Antagonism in HIV Infection: Current Concepts and Future Opportunities. Annu Rev Med 63: 15.11–15.13. 10.1146/annurev-med-052010-145454 PMC3298858 22034870

[5] Klein RS (2008) A moving target: the multiple roles of CCR5 in infectious diseases. J Infect Dis 197: 183–186. 18179384 10.1086/524692

[6] Lim JK, Glass WG, McDermott DH, Murphy PM (2006) CCR5: no longer a “good for nothing” gene–chemokine control of West Nile virus infection. Trends Immunol 27: 308–312. 16753343 10.1016/j.it.2006.05.007

[7] Glass WG, Lim JK, Cholera R, Pletnev AG, Gao JL, et al (2005) Chemokine receptor CCR5 promotes leukocyte trafficking to the brain and survival in West Nile virus infection. J Exp Med 202: 1087–1098. 16230476 10.1084/jem.20042530 PMC2213214

[8] Glass WG, McDermott DH, Lim JK, Lekhong S, Yu SF, et al (2006) CCR5 deficiency increases risk of symptomatic West Nile virus infection. J Exp Med 203: 35–40. 16418398 10.1084/jem.20051970 PMC2118086

[9] Lim JK, McDermott DH, Lisco A, Foster GA, Krysztof D, et al (2010) CCR5 deficiency is a risk factor for early clinical manifestations of West Nile virus infection but not for viral transmission. J Infect Dis 201: 178–185. 20025530 10.1086/649426 PMC2934858

[10] Kindberg E, Mickiene A, Ax C, Akerlind B, Vene S, et al (2008) A deletion in the chemokine receptor 5 (CCR5) gene is associated with tickborne encephalitis. J Infect Dis 197: 266–269. 18179389 10.1086/524709

[11] Pulendran B, Miller J, Querec TD, Akondy R, Moseley N, et al (2008) Case of yellow fever vaccine–associated viscerotropic disease with prolonged viremia, robust adaptive immune responses, and polymorphisms in CCR5 and RANTES genes. J Infect Dis 198: 500–507. 18598196 10.1086/590187 PMC3734802

[12] Gould EA, Solomon T (2008) Pathogenic flaviviruses. Lancet 371: 500–509. 18262042 10.1016/S0140-6736(08)60238-X

[13] Solomon T (2004) Flavivirus encephalitis. N Engl J Med 351: 370–378. 15269317 10.1056/NEJMra030476

[14] Halstead SB, Jacobson J (2003) Japanese encephalitis. Adv Virus Res 61: 103–138. 14714431 10.1016/s0065-3527(03)61003-1

[15] Larena M, Lobigs M (2011) Immunobiology of Japanese Encephalitis Virus, Flavivirus Encephalitis, Daniel Růz˘ek, editors. ISBN: 978-953-307-669-0, InTech. Available: http://www.intechopen.com/articles/show/title/immunobiology-of-japanese-encephalitis-virus.

[16] Larena M, Regner M, Lee E, Lobigs M (2011) Pivotal role of antibody and subsidiary contribution of CD8+ T cells to recovery from infection in a murine model of Japanese encephalitis. J Virol 85: 5446–5455. 21450826 10.1128/JVI.02611-10 PMC3094953

[17] Lee E, Lobigs M (2002) Mechanism of virulence attenuation of glycosaminoglycan-binding variants of Japanese encephalitis virus and Murray Valley encephalitis virus. J Virol 76: 4901–4911. 11967307 10.1128/JVI.76.10.4901-4911.2002 PMC136177

[18] Kiessling R, Klein E, Wigzell H (1975) “Natural” killer cells in the mouse. I. Cytotoxic cells with specificity for mouse Moloney leukemia cells. Specificity and distribution according to genotype. Eur J Immunol 5: 112–117. 1234049 10.1002/eji.1830050208

[19] Licon Luna RM, Lee E, Mullbacher A, Blanden RV, Langman R, et al (2002) Lack of both Fas ligand and perforin protects from flavivirus-mediated encephalitis in mice. J Virol 76: 3202–3211. 11884544 10.1128/JVI.76.7.3202-3211.2002 PMC136025

[20] Kuziel WA, Dawson TC, Quinones M, Garavito E, Chenaux G, et al (2003) CCR5 deficiency is not protective in the early stages of atherogenesis in apoE knockout mice. Atherosclerosis 167: 25–32. 12618265 10.1016/s0021-9150(02)00382-9

[21] Lee E, Hall RA, Lobigs M (2004) Common E protein determinants for attenuation of glycosaminoglycan-binding variants of Japanese encephalitis and West Nile viruses. J Virol 78: 8271–8280. 15254199 10.1128/JVI.78.15.8271-8280.2004 PMC446099

[22] Tscharke DC, Karupiah G, Zhou J, Palmore T, Irvine KR, et al (2005) Identification of poxvirus CD8+ T cell determinants to enable rational design and characterization of smallpox vaccines. J Exp Med 201: 95–104. 15623576 10.1084/jem.20041912 PMC2212779

[23] Momburg F, Mullbacher A, Lobigs M (2001) Modulation of transporter associated with antigen processing (TAP)-mediated peptide import into the endoplasmic reticulum by flavivirus infection. J Virol 75: 5663–5671. 11356974 10.1128/JVI.75.12.5663-5671.2001 PMC114279

[24] Colombage G, Hall R, Pavy M, Lobigs M (1998) DNA-based and alphavirus-vectored immunisation with prM and E proteins elicits long-lived and protective immunity against the flavivirus, Murray Valley encephalitis virus. Virology 250: 151–163. 9770429 10.1006/viro.1998.9357

[25] Lobigs M, Pavy M, Hall RA, Lobigs P, Cooper P, et al (2010) An inactivated Vero cell-grown Japanese encephalitis vaccine formulated with Advax, a novel inulin-based adjuvant, induces protective neutralizing antibody against homologous and heterologous flaviviruses. J Gen Virol 91: 1407–1417. 20130134 10.1099/vir.0.019190-0 PMC2888167

[26] Kesson AM, Blanden RV, Mullbacher A (1987) The primary in vivo murine cytotoxic T cell response to the flavivirus, West Nile. J Gen Virol 68 Pt 7:2001–2006. 3496425 10.1099/0022-1317-68-7-2001

[27] Purtha WE, Myers N, Mitaksov V, Sitati E, Connolly J, et al (2007) Antigen-specific cytotoxic T lymphocytes protect against lethal West Nile virus encephalitis. Eur J Immunol 37: 1845–1854. 17559174 10.1002/eji.200737192

[28] Hardison JL, Wrightsman RA, Carpenter PM, Kuziel WA, Lane TE, et al (2006) The CC chemokine receptor 5 is important in control of parasite replication and acute cardiac inflammation following infection with Trypanosoma cruzi. Infect Immun 74: 135–143. 16368966 10.1128/IAI.74.1.135-143.2006 PMC1346647

[29] Khan IA, Thomas SY, Moretto MM, Lee FS, Islam SA, et al (2006) CCR5 is essential for NK cell trafficking and host survival following Toxoplasma gondii infection. PLoS Pathog 2: 484–500. 10.1371/journal.ppat.0020049 PMC1475660 16789839

[30] Dawson TC, Beck MA, Kuziel WA, Henderson F, Maeda N (2000) Contrasting effects of CCR5 and CCR2 deficiency in the pulmonary inflammatory response to influenza A virus. Am J Pathol 156: 1951–1959. 10854218 10.1016/S0002-9440(10)65068-7 PMC1850091

[31] Fadel SA, Bromley SK, Medoff BD, Luster AD (2008) CXCR3-deficiency protects influenza-infected CCR5-deficient mice from mortality. Eur J Immunol 38: 3376–3387. 19039768 10.1002/eji.200838628 PMC2749081

[32] Kohlmeier JE, Miller SC, Smith J, Lu B, Gerard C, et al (2008) The chemokine receptor CCR5 plays a key role in the early memory CD8+ T cell response to respiratory virus infections. Immunity 29: 101–113. 18617426 10.1016/j.immuni.2008.05.011 PMC2519120

[33] Thapa M, Kuziel WA, Carr DJ (2007) Susceptibility of CCR5-deficient mice to genital herpes simplex virus type 2 is linked to NK cell mobilization. J Virol 81: 3704–3713. 17267483 10.1128/JVI.02626-06 PMC1866094

[34] Olive AJ, Gondek DC, Starnbach MN (2011) CXCR3 and CCR5 are both required for T cell-mediated protection against C. trachomatis infection in the murine genital mucosa. Mucosal Immunol 4: 208–216. 20844481 10.1038/mi.2010.58 PMC3010299

[35] Algood HM, Flynn JL (2004) CCR5-deficient mice control Mycobacterium tuberculosis infection despite increased pulmonary lymphocytic infiltration. J Immunol 173: 3287–3296. 15322191 10.4049/jimmunol.173.5.3287

[36] Ank N, Petersen K, Malmgaard L, Mogensen SC, Paludan SR (2005) Age-dependent role for CCR5 in antiviral host defense against herpes simplex virus type 2. J Virol 79: 9831–9841. 16014944 10.1128/JVI.79.15.9831-9841.2005 PMC1181601

[37] Nansen A, Christensen JP, Andreasen SO, Bartholdy C, Christensen JE, et al (2002) The role of CC chemokine receptor 5 in antiviral immunity. Blood 99: 1237–1245. 11830471 10.1182/blood.v99.4.1237

[38] Peterson KE, Errett JS, Wei T, Dimcheff DE, Ransohoff R, et al (2004) MCP-1 and CCR2 contribute to non-lymphocyte-mediated brain disease induced by Fr98 polytropic retrovirus infection in mice: role for astrocytes in retroviral neuropathogenesis. Journal of virology 78: 6449–6458. 15163738 10.1128/JVI.78.12.6449-6458.2004 PMC416512

[39] Zhong MX, Kuziel WA, Pamer EG, Serbina NV (2004) Chemokine receptor 5 is dispensable for innate and adaptive immune responses to Listeria monocytogenes infection. Infection and immunity 72: 1057–1064. 14742553 10.1128/IAI.72.2.1057-1064.2004 PMC321636

[40] Aliberti J, Reis e Sousa C, Schito M, Hieny S, Wells T, et al (2000) CCR5 provides a signal for microbial induced production of IL-12 by CD8 alpha+ dendritic cells. Nat Immunol 1: 83–87. 10881180 10.1038/76957

[41] Castellino F, Huang AY, Altan-Bonnet G, Stoll S, Scheinecker C, et al (2006) Chemokines enhance immunity by guiding naive CD8+ T cells to sites of CD4+ T cell-dendritic cell interaction. Nature 440: 890–895. 16612374 10.1038/nature04651

[42] Hickman HD, Li L, Reynoso GV, Rubin EJ, Skon CN, et al (2011) Chemokines control naive CD8+ T cell selection of optimal lymph node antigen presenting cells. J Exp Med 208: 2511–2524. 22042976 10.1084/jem.20102545 PMC3256957

[43] Hugues S, Scholer A, Boissonnas A, Nussbaum A, Combadiere C, et al (2007) Dynamic imaging of chemokine-dependent CD8+ T cell help for CD8+ T cell responses. Nat Immunol 8: 921–930. 17660821 10.1038/ni1495

[44] Floto RA, MacAry PA, Boname JM, Mien TS, Kampmann B, et al (2006) Dendritic cell stimulation by mycobacterial Hsp70 is mediated through CCR5. Science 314: 454–458. 17053144 10.1126/science.1133515

[45] Kohlmeier JE, Reiley WW, Perona-Wright G, Freeman ML, Yager EJ, et al (2011) Inflammatory chemokine receptors regulate CD8(+) T cell contraction and memory generation following infection. J Exp Med 208: 1621–1634. 21788409 10.1084/jem.20102110 PMC3149221

[46] Belnoue E, Kayibanda M, Deschemin JC, Viguier M, Mack M, et al (2003) CCR5 deficiency decreases susceptibility to experimental cerebral malaria. Blood 101: 4253–4259. 12560237 10.1182/blood-2002-05-1493

[47] Souza AL, Souza PR, Pereira CA, Fernandes A, Guabiraba R, et al (2011) Experimental infection with Schistosoma mansoni in CCR5-deficient mice is associated with increased disease severity, as CCR5 plays a role in controlling granulomatous inflammation. Infect Immun 79: 1741–1749. 21263020 10.1128/IAI.00502-10 PMC3067544

[48] Sato N, Kuziel WA, Melby PC, Reddick RL, Kostecki V, et al (1999) Defects in the generation of IFN-gamma are overcome to control infection with Leishmania donovani in CC chemokine receptor (CCR) 5-, macrophage inflammatory protein-1 alpha-, or CCR2-deficient mice. J Immunol 163: 5519–5525. 10553079

[49] Huffnagle GB, McNeil LK, McDonald RA, Murphy JW, Toews GB, et al (1999) Cutting edge: Role of C-C chemokine receptor 5 in organ-specific and innate immunity to Cryptococcus neoformans. J Immunol 163: 4642–4646. 10528159

[50] Komatsu K, Miyazaki D, Morohoshi K, Kuo CH, Kakimaru-Hasegawa A, et al (2008) Pathogenesis of herpetic stromal keratitis in CCR5- and/or CXCR3-deficient mice. Curr Eye Res 33: 736–749. 18798077 10.1080/02713680802344716

[51] Glass WG, Lane TE (2003) Functional expression of chemokine receptor CCR5 on CD4(+) T cells during virus-induced central nervous system disease. J Virol 77: 191–198. 12477824 10.1128/JVI.77.1.191-198.2003 PMC140629

[52] Salazar-Gonzalez JF, Salazar MG, Keele BF, Learn GH, Giorgi EE, et al (2009) Genetic identity, biological phenotype, and evolutionary pathways of transmitted/founder viruses in acute and early HIV-1 infection. J Exp Med 206: 1273–1289. 19487424 10.1084/jem.20090378 PMC2715054

[53] Weiss ID, Shoham H, Wald O, Wald H, Beider K, et al (2011) Ccr5 deficiency regulates the proliferation and trafficking of natural killer cells under physiological conditions. Cytokine 54: 249–257. 21376626 10.1016/j.cyto.2011.01.011

[54] Lobigs M, Blanden RV, Mullbacher A (1996) Flavivirus-induced up-regulation of MHC class I antigens; implications for the induction of CD8+ T-cell-mediated autoimmunity. Immunol Rev 152: 5–19. 8930665 10.1111/j.1600-065X.1996.tb00908.x PMC7165549

[55] Kesson AM, King NJ (2001) Transcriptional regulation of major histocompatibility complex class I by flavivirus West Nile is dependent on NF-kappaB activation. J Infect Dis 184: 947–954. 11574908 10.1086/323603

[56] King NJ, Kesson AM (1988) Interferon-independent increases in class I major histocompatibility complex antigen expression follow flavivirus infection. J Gen Virol 69 Pt 10:2535–2543. 2844965 10.1099/0022-1317-69-10-2535

[57] Lobigs M, Mullbacher A, Regner M (2003) MHC class I up-regulation by flaviviruses: Immune interaction with unknown advantage to host or pathogen. Immunol Cell Biol 81: 217–223. 12752686 10.1046/j.1440-1711.2003.01161.x

[58] Mullbacher A, Lobigs M (1995) Up-regulation of MHC class I by flavivirus-induced peptide translocation into the endoplasmic reticulum. Immunity 3: 207–214. 7544229 10.1016/1074-7613(95)90090-x

[59] Shrestha B, Samuel MA, Diamond MS (2006) CD8+ T cells require perforin to clear West Nile virus from infected neurons. J Virol 80: 119–129. 16352536 10.1128/JVI.80.1.119-129.2006 PMC1317548

[60] Erlanger TE, Weiss S, Keiser J, Utzinger J, Wiedenmayer K (2009) Past, present, and future of Japanese encephalitis. Emerg Infect Dis 15: 1–7. 19116041 10.3201/eid1501.080311 PMC2660690

[61] Simon V, Ho DD, Abdool Karim Q (2006) HIV/AIDS epidemiology, pathogenesis, prevention, and treatment. Lancet 368: 489–504. 16890836 10.1016/S0140-6736(06)69157-5 PMC2913538

